# Incidence Patterns and Temporal Trends of Invasive Nonmelanotic Vulvar Tumors in Germany 1999-2011. A Population-Based Cancer Registry Analysis

**DOI:** 10.1371/journal.pone.0128073

**Published:** 2015-05-28

**Authors:** Nina Buttmann-Schweiger, Stefanie J. Klug, Alexander Luyten, Bernd Holleczek, Florian Heitz, Andreas du Bois, Klaus Kraywinkel

**Affiliations:** 1 Robert Koch-Institute, Department of Epidemiology and Health Monitoring, German Centre for Cancer Registry Data, Berlin, Germany; 2 Cancer Epidemiology, University Cancer Centre Dresden, University Hospital Carl Gustav Carus, Technische Universität Dresden, Dresden, Germany; 3 Department of Gynaecology and Obstetrics, Klinikum Wolfsburg, Wolfsburg, Germany; 4 Saarland Cancer Registry, Saarbrücken, Germany; 5 Department of Gynecology and Gynecologic Oncology, Kliniken Essen Mitte, Essen, Germany; Rudjer Boskovic Institute, CROATIA

## Abstract

**Objectives:**

Time trends on the incidence and characteristics of invasive vulvar cancer in Germany have so far been studied in few local population- and hospital based tumor registries. We aimed to provide an overview on recent developments of vulvar cancer in Germany, using population-based cancer registry data.

**Methods:**

We analyzed the data on vulvar cancer of eight population-based German cancer registries for the period 1999-2011. ICD-10 codes and ICD-O-3 morphology codes were used to select site and histologic types. The annual percentage change was calculated on age-adjusted incidence rates with a joinpoint regression model.

**Results:**

A total of 12,711 registered cases of invasive carcinoma of the vulva were included in the analyses, hereof were 12,205 of squamous cell origin. Age-standardized incidence rates of vulvar cancer annually increased by 6.7% (95% confidence limits: 5.6-7.9) from 1.7 per 100,000 women in 1999 to 3.6 per 100,000 women in 2011. An increase was observed among women of all ages, and especially between 30 and 69 years of age.

**Conclusion:**

The annual incidence of invasive carcinoma of the vulva nearly doubled in the past decade in Germany, considerably exceeding the rates observed in other Western European countries. A combination of changes in risk factors, and documentation practice might have contributed to the observed substantial increase in vulvar cancer incidence.

## Introduction

Vulvar carcinoma is a rare malignancy, with an estimated age-standardized (world standard) incidence rate up to 1.5 women per 100,000 women per year worldwide as it has also been described for Eastern Germany in the late 1970/1980’s and early 2000’s [1,2]. An analysis based on two western German federal population-based cancer registries estimated 2000 to 2700 new cases per year in Germany for the time period 2000–2004 [3]. A recent report based on more population-based cancer registries in Germany estimated around 3200 cases for the year 2010. The incidence increases with age, the highest burden of disease is accounted for by women over 70 years of age in Germany [4].

The most common malignant tumor of the vulva is of squamous cell origin (SCC) and can etiologically be classified in two main subtypes: potentially associated with human papillomavirus (HPV) are warty and basaloid SCC, preceded by histologically corresponding intraepithelial lesions (VIN), while the non-HPV associated keratinizing SCC type is associated with lichen sclerosus [5]. The latter is found predominantly in older women, whereas HPV related vulvar cancer is more common in younger age [6]. Concomitant risk factors to HPV infection are smoking and a status of immunosuppression as observed in HIV infected women and in transplant patients [7–12].

Invasive vulvar cancer has been reported to be increasingly diagnosed in clinical settings in Germany [13,14]. In a few other countries an increase of vulvar cancer has also been reported [15–19]. We present the age-adjusted and age-specific incidence rates and the development of different tumor-characteristics of invasive vulvar cancer in Germany during the past decade by analyzing population based cancer registry data.

## Methods

The database used in the present analysis was obtained from the German Centre for Cancer Registry Data at the Robert Koch Institute (‘Zentrum für Krebsregisterdaten’, ZfKD), which annually collects anonymized incidence and survival data from all federal states’ cancer registries. Continuous population based cancer registration is being performed since 1967 in the federal state of Saarland, while the majority of registries in Germany started around the year 2000, and nationwide coverage was reached in 2009. Patients consent was not obtained, since only anonymized data is available for analysis. Data are available only upon request because of a legal restriction. Please see the Data Availability Statement for further details.

For the analyses performed in this article, we included the anonymized cancer data of patients diagnosed with invasive vulvar carcinoma (coded as C51 according to the International Statistical Classification of Diseases and Related Health Problems 10th Revision (ICD-10)) from eight population-based German cancer registries, covering approximately 60% of the German female population for the time period of 2003–2011 in the federal states of Bavaria, Berlin, Brandenburg, Bremen, Hamburg, Lower Saxony, Mecklenburg-W. Pomerania, Saxony, Saxony-Anhalt, Schleswig-Holstein, the Saarland, Thuringia, and in the administrative district of Münster. From six of the above mentioned registries, additional data for the years 1999–2002 were available. In addition, Saarland and Hamburg contributed data from 1991 onwards. Cases notified by death certificate only (DCO) and cancers without further specification of tumor morphology were studied separately.

The subsites of tumor origin were categorized into ‘Subsite specified’ (C51.0-C51.8), ‘Labiae’ (combining labiae majores et minores C51.0, C51.1), ‘Clitoris’ (C51.2), ‘Overlapping’ (C51.8), and ‘Subsite not specified’ (C51.9).

Morphology codes were used to determine histologic subgroups according to the International Classification of Diseases for Oncology (ICD-O-3). Invasive malignant tumors were excluded from the main analyses if they were not further specified (morphology codes 8000–8046), or if they were malignant melanoma (8720–8790). Groups were classified according to ‘WHO classification of tumors of the vulva’ as ‘squamous cell carcinoma (SCC)’ (8050–8084, 8090–8110) [20]; with a further differentiation into: ‘keratinizing SCC’ (8071), ‘non-keratinizing / basaloid-warty / verrucous SCC’ (8051, 8072, 8083), and ‘SCC tumors not otherwise specified’ (8070); as well as ‘epithelial tumors other than SCC and non-epithelial tumors’ (8120–8710, 8800–9581).

Staging of disease was categorized as T1 (localized, ≤2cm in greatest dimension), T2 (localized, >2cm in greatest dimension), and T3/T4 (regional and locally advanced, infiltrating the urethra, vagina, anus or the surrounding tissue, mucosa of the bladder, rectum, bound to pelvic bone), using the T-classifications of malignant tumors editions 4–6. Major changes in the classification system of tumor stages were introduced with the 7th edition of the TNM—classification in 2010 [21–23]. Due to missing information on the edition used in some of the registries, the years 2010 and 2011 were excluded from the analyses on tumor stage. Sparse data on lymph node- and distant metastasis limited further analyses according to clinical stage groups.

Annual age-standardized (European standard) incidence rates per 100 000 women (ASIR) were calculated for all ages combined and truncated ASIR were derived for the age groups 30 to 49 years, 50 to 69 years and 70 years and older, respectively. A standardized rate ratio and its exact 95% confidence interval were calculated according to IARC principles and methods [24]. The average annual percentage change (AAPC) was calculated with regression models using ‘Joinpoint’, a software developed by the United States National Cancer Institute to test whether changes in trends were statistically significantly different from zero (alpha <.05) [25].

Incidence rates of cervical adenocarcinoma (C53 with morphology codes 8140–8384) and endometrial cancer (C54, C55) were calculated equivalently to the vulvar cancer rates. Cervical adenocarcinoma potentially shares the same etiology with a proportion of vulva carcinomas among younger patients, namely involvement of HPV infection; the impact of screening on incidence rates, however, is smaller compared to cervical SCC. Endometrial tumors are diagnosed across an age distribution similar to non HPV-induced vulvar cancer. These two cancers are usually treated within the same medical specialty (gynecology) and reported by the same physicians. Thus they present an opportunity to examine potential systematic detection or documentation bias.

For a comparison of German incidence data with those from other European countries, we included DCO notified cases and cases with unspecified morphology in the German figures, and retrieved figures on invasive vulvar cancer from selected cancer registries in Europe. As vulvar cancer is neither depicted as a separate entity in the datasources ‘Globocan 2012’ or in ‘Cancer Incidence in Five Continents’ [6,26], cancer registries with nationwide coverage, a sufficient degree of completeness, according to the International Agency for Research on Cancer (IARC) [6], and with geographic proximity to Germany that provided vulvar cancer incidence rates were selected: Austria, Belgium, the Czech Republic, the Netherlands, Norway, Sweden, and the United Kingdom.

## Results

**Fig 1:**
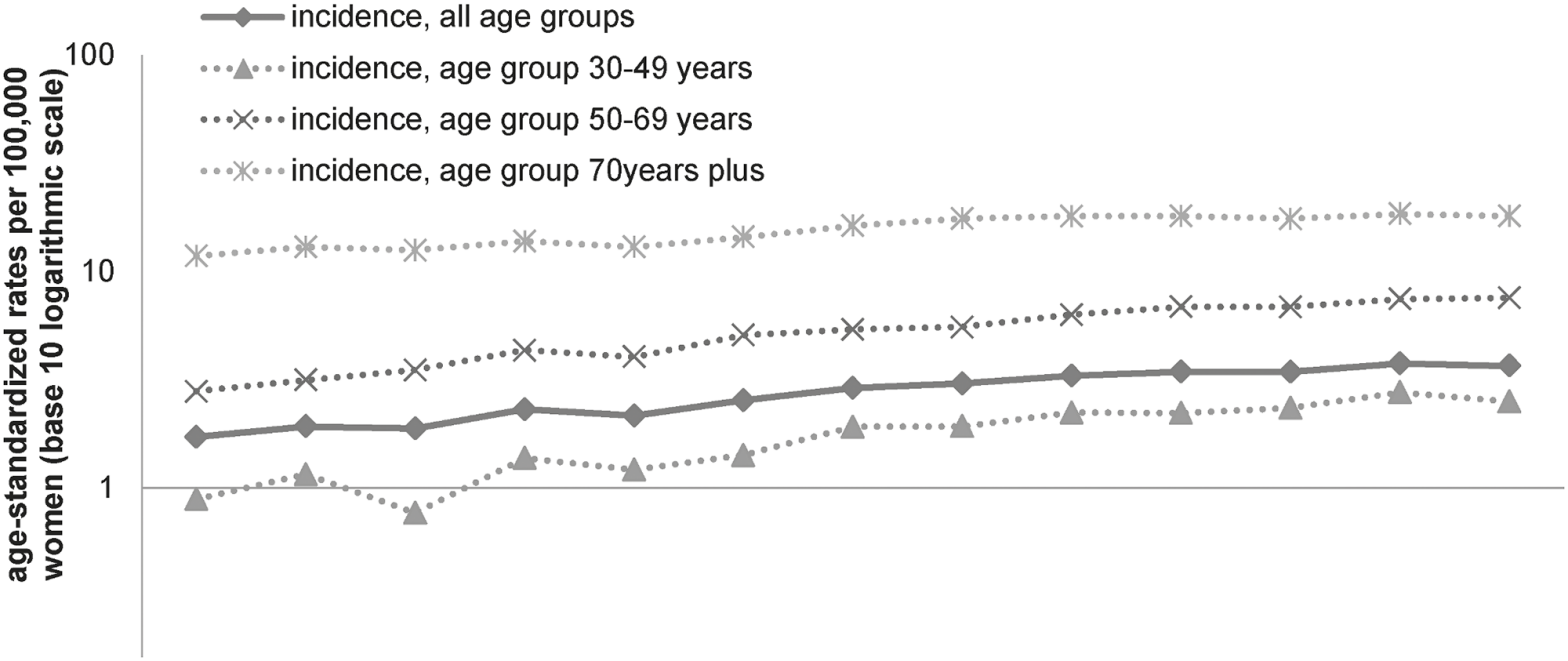
Vulvar cancer incidence rates from 1999–2011, stratified by age groups. Annual age-standardized vulvar cancer incidence rates per 100,000 women (old European Standard on a base-10 logarithmic scale), by calendar year, all ages (straight line), and for the age groups '30–49 years', '50–69 years', and '70 years plus' (dashed lines); DCO notified cases, histologically not further specified cases and melanoma excluded.

An overall number of 14,637 women with invasive vulvar cancer were observed whereof 12,711 cases (86.8%) were valid for further analyses. In total, 905 DCO cases, 607 non-DCO cases with unspecified histology, and 414 malignant melanoma were excluded from the main analysis. Median age of the included patients dropped from 73 years in 1999–2002 to 71 years in 2007–2011. Age-standardized incidence rates (ASIR) of invasive vulvar cancer annually increased by 6.7% (95%CI 5.6–7.9) from 1.7 per 100,000 in 1999 to 3.7 per 100,000 in 2011, corresponding to a standardized rate ratio of 2.1 (95%CI 1.9–2.4). All age groups displayed this trend (Fig 1). A decrease in the proportions of DCO notified cancers and of histologically not further specified carcinomas was observed over time. Before exclusion of DCO notified cases, and histologically not further specified carcinoma from the analyses, vulvar cancer annually increased by 5.8% (95%CI 4.8–6.8).

Overall, 55.7% of the women were diagnosed at the age of 70 years and above, 30.8% at the age of 50–69 years and 11.3% at the age of 30–49 years. Statistically significant annual increases in ASIR were observed in all age groups, however most pronounced in women aged 30–49 years (AAPC +9.7%) (Table 1). The most frequently observed histological subtype was SCC (96.0%), whereof the keratinizing type predominated in 52.4% of cases. 506 cases were of non-epithelial origin (4.0%). A significant annual increase of SCC tumors in general was observed, whereas the increase in the incidence rate of epithelial tumors with no SCC origin and non-epithelial tumors remained non-significant. In 60.9% of the cases, a specific subsite was not provided. Of all specified subsites, labial and overlapping lesions were most common, and in 15.2% percent of cases vulvar tumors were diagnosed at the clitoris. Significant annual increases in the ASIR for all subsites were observable, most pronounced for clitoral tumors (AAPC +11.7%) (Table 1). Nine out of ten women were diagnosed with a tumor staged as ‘T1’, however, the proportion of cases with unspecified or missing information was substantial (17.3%). We observed significant annual increases across all stages, foremost in small T1 tumors (Table 1).

**Table 1:** Women’s age at diagnosis and characteristics of the invasive vulvar tumors diagnosed from 1999–2011 (for topography up to 2009).

Characteristics	N	AAPC of ASIR^a^ (95% CI)	
**Age at diagnosis**			
Overall	12711	6.7	(5.6–7.9)
30–49 years	1441	9.7	(7.6–11.9)
50–69 years	3921	8.0	(6.7–9.3)
70+ years	7084	3.8	(2.7–4.9)
**Morphology**			
Squamous cell carcinoma (SCC)	12205	6.8	(5.8–7.9)
SCC tumors not otherwise specified	4114	8.2	(7.0–9.5)
Keratinizing SCC	6392	6.4	(5.0–7.8)
Non-keratinizing / basaloid-warty / verrucous SCC	869	6.1	(3.7–8.5)
Epithelial tumors other than SCC and non-epithelial tumors	506	3.6	((-0.1)-7.5)
**Subsite**			
Subsite specified	4975	6.9	(4.6–9.2)
Labiae	2742	5.3	(3.2–7.4)
Clitoris	756	11.7	(7.6–15.8)
Overlapping	1477	6.6	(3.5–9.8)
Subsite not specified	7736	6.5	(5.2–7.8)
**Staging**			
Staging specified	8104	8.2	(7.1–9.4)
T1	3774	9.2	(7.9–10.6)
T2	3619	7.3	(5.5–9.1)
T3/T4	711	5.8	(2.4–9.2)
Staging not specified	1690	3.8	(0.6–7.2)

Staging limited to data from 1999–2009 (TNM editions 4–6).

^a^The average annual percentage change (AAPC) from 1999–2011 of the age standardized (old European standard) incidence rate (ASIR).

Additional information on age specific trends over the time periods 1999–2002, 2003–2006 and 2007–2011, as well as on cervical adenocarcinoma and endometrial carcinoma incidence is provided in the supplement. Briefly, in patients below the age of 70 years, the increase of SCC cases and tumors without information on subsite were significantly more pronounced. Tumors staged as T1 and T2 significantly increased in all age groups, whereas regional and locally advanced tumors (T3/T4) significantly increased only in women below 70 years of age (S1 Table). No changes in the annual age-standardized incidence rates of cervical adenocarcinoma and carcinoma of the uterus were observed (S1 Fig).

**Fig 2:**
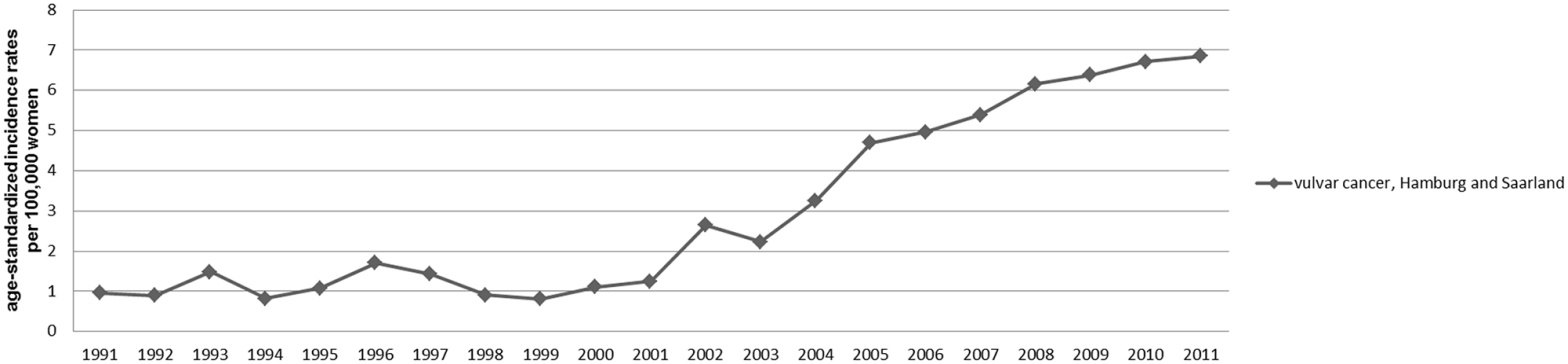
Vulvar cancer incidence rates in Hamburg and Saarland from 1991–2011. Annual age-standardized vulvar cancer incidence rates per 100,000 women (old European Standard) by calendar year, all ages; DCO notified cases, histologically not further specified cases and melanoma excluded.

Additionally, results based on data of the registries of Hamburg and Saarland indicated that the observed increase of the incidence of invasive vulva cancers represents an ongoing development, that had already started in the early 2000s (Fig 2).

Age-standardized incidence rates from the Netherlands and from Belgium showed an increase in vulvar cancer incidence, whereas results from other European cancer registry data suggested no change in disease burden (Table 2). In 2010/2011, the age-standardized incidence rate in Germany was 1.2-fold to 2.2-fold higher than the rates in other European countries.

**Table 2:** Invasive vulvar cancer incidence rates from 1999–2011 in selected countries. DCO cases, melanoma, and histologically not further specified cases included.

Countries	References	1999	2000	2001	2002	2003	2004	2005	2006	2007	2008	2009	2010	2011
**Austria**	Statistic Austria, Austrian national cancer registry, personal communication	2.0	2.0	2.1	2.0	2.1	2.2	2.1	2.2	2.1	2.3	1.8	2.2	1.9
**Belgium**	http://www.kankerregister.org	1.7^a^	1.8^a^	1.6^a^	1.9^a^	1.9	1.9	2.1	2.1	2.1	2.2	2.3	2.6	2.2
**Sweden**	http://www.socialstyrelsen.se/statistik/statistikdatabas/cancer	1.8	1.8	2.0	1.5	1.7	1.7	1.8	1.9	2.1	1.8	1.5	1.9	1.8
**Netherlands**	http://www.cijfersoverkanker.nl/home-36.html	-	-	2.2	2.1	2.4	2.3	2.4	2.4	2.8	2.7	3.1	3.0	3.4
**Norway**	Cancer Registry of Norway, personal communication	2.3	1.6	2.7	1.6	2.6	2.5	3.1	2.8	2.7	2.1	2.4	2.3	2.3
**UK**	http://www.cancerresearchuk.org/cancer-info/cancerstats/types/	2.4	2.2	2.5	2.3	2.5	2.3	2.4	2.5	2.5	2.6	2.6	2.6	2.5
**Czech Republic**	http://www.svod.cz	2.9	3.0	2.2	2.6	2.6	2.7	2.9	2.6	2.4	2.5	2.5	2.7	-
***Germany***	***German cancer registries used in this article***	*2*.*2*	*2*.*4*	*2*.*3*	*2*.*7*	*2*.*6*	*2*.*9*	*3*.*2*	*3*.*4*	*3*.*7*	*3*.*8*	*3*.*7*	*4*.*1*	*4*.*0*

Numbers represent annual age-standardized incidence rates per 100,000 women (old European Standard) of invasive vulvar cancer in selected national population-based cancer registries.

^a^Region Flamish only.

## Discussion

According to data from German cancer registries, the age-standardized incidence rate of invasive carcinoma of the vulva in Germany almost doubled over the past twelve years. This trend was present in women of all age groups, however most pronounced among women aged 30–49 years and 50–69 years. As a result, the proportion of younger patients slightly increased despite reciprocal changes in German population. Our results, using data from Eastern and Western German population-based cancer registries, support observations from hospital-based studies in local Western German settings of recently increasing vulvar cancer burden in Germany [12, 13]. A rise has not been observed in a preceding population- based cancer registry study from Eastern Germany comparing data from the years 1998–2002 with data from the years 1976–1989 [2]. In previous studies, a moderate increase of invasive vulvar tumors across all age groups was described for the US [27], and significant increases for Canada (especially in the oldest age group) and the UK [18,19]. In recent Danish and Dutch registry-based studies, as well as in an Austrian retrospective study using data from 1985–1988 and 1994–1997, an increase predominantly was observed in younger women [15–17]. The assessment of data from other European cancer registries provided another source for comparison of trends in vulvar cancer incidence rates on the general population level. Moderately rising trends over the last decade were observed in Belgium and the Netherlands, whereas there was no evidence for increasing rates in other European regions. Increasing rates described in the aforementioned Austrian study [17] did not continue in the Austrian cancer registry data after 1999. In recent years, the burden from vulvar cancer in Germany was considerably higher than in other European countries.

The potential of documentation bias is a major concern in analyses of cancer registry data. Despite of declining proportions of DCO notified cases and histologically not further specified cases, inclusion of these cases had only little effect on the annual percentage changes of the vulvar cancer rates.—In addition, the largest increase was observed in Hamburg and Saarland, two federal states with a long history of cancer registration, and a high data quality [26]. Furthermore, the observed trends of the incidence rates of endometrial cancer during the study period suggest that artifacts from changed registration might not be a major cause for the observed findings. Theoretically, it could also be that decreasing rates of endometrial cancer did not become apparent due to a concurrent trend of intensified documentation or registration. We are not aware of any indication however, that selected increased registration of gynecological tumors had occurred. An increase of the age-standardized vulvar cancer mortality rates in Germany from 0.7 per 100,000 women in 1999–2002 to 0.9 per 100,000 women in 2009–2012 [28] further supports our findings of an increase of vulvar cancer over the last decade.

Increasing incidence rates in some cancers may also be explained by improved diagnostic procedures, which has been observed in thyroid cancer [29], or by increased awareness of the disease. However, the observed continuous increase in incidence rates across all T-stages suggests that improved awareness and diagnosis cannot entirely explain the observed trends.

Hence, an increasing prevalence of risk factors in the population has to be considered. About 25% of vulvar cancer worldwide is assumed to be etiologically linked to HPV infection [30]. A more permissive sexual behavior in German female birth cohorts from 1940 onwards [31] might have led to a higher HPV prevalence and possibly resulted in more cases of vulvar cancer. On the other hand, incidence rates of cervical adenocarcinoma, a largely HPV related disease, have not shown marked increases in the German registries over the same time period. Furthermore, a genital high risk HPV prevalence of 6.3% in the German female population aged 30 years and above is not higher than in neighboring European populations [32–34]. The difference between German vulvar cancer incidence rates and those of other European countries remains therefore unexplained.

Smoking is considered another independent risk factor for the development of vulvar cancer [7,8]. Concurrent to an increase in smoking prevalence in German women, the age of smoking initiation dropped [35,36]. The strong increase in women’s tobacco consumption in Germany in female birth cohorts 1930–1959 from about 30% to more than 60% of women ever having smoked (health survey 2003) might have contributed to the observed increase in vulvar cancer incidence. However, a similar increase of smoking prevalence is observed in most Western countries, seemingly without much impact on vulva cancer rates. Lung cancer in German women has increased by about 30% over the study period 1999–2011 [37] and one would expect the effect of smoking on vulva cancer incidence to be less pronounced than on lung cancer, where smoking is the predominant risk factor. Lastly, women with immunodeficiency, e.g. due to organ transplantation or HIV infection have a substantial excess risk for vulvar cancer [9–12]. Organ transplantation and HIV are fairly rare in Germany, but the increase in organ transplantation since 1997 [38], together with an increasing number of women living with HIV [39], might have also contributed to the increase in the vulvar cancer burden in Germany. Major strengths of our investigation are the representativeness of results for Germany, and the large number of cases (>14,000) of this relatively rare disease, due to the large size of the female population covered. Missing values in staging and sublocations however limit the interpretation of some of the results.

## Conclusion

A doubling of incidence rates for vulvar cancer should not be ignored from an etiological perspective, even if vulvar cancer remains a rare disease accounting for 1.7 to 3.6 new cases per calendar year in 100,000 women. A combination of risk factors (HPV, smoking, sexual behavior, HIV, organ transplantation), and documentation practice might have contributed to the observed substantial increase in vulvar cancer incidence, however there is only evidence on substantial increases in the prevalence of smoking in the German female population.

## Supporting Information

S1 Fig Incidence rates of cervical adenocarcinoma and cancer of the uterus from 1999–2011. Annual age-standardized incidence rates per 100,000 women (old European Standard on a base-10 logarithmic scale) of cervical adenocarcinoma and cancer of the uterus, 1999–2011.
(TIF)

S1 Table Average annual percentage changes of vulvar cancer incidence rates from 1999–2011. Average annual percentage changes (AAPC) and corresponding 95% confidence intervals (95%CI) from 1999–2011 of age-standardized vulvar cancer incidence rates per 100,000 women (old European Standard), stratified by the tumor characteristics morphology, topography, and staging in specific age groups.
(XLSX)
