# Supplementary figures and images for: Incidence Patterns and Temporal Trends of Invasive Nonmelanotic Vulvar Tumors in Germany 1999-2011. A Population-Based Cancer Registry Analysis
Source: PLoS One. 2015 May 28;10(5):e0128073. doi: 10.1371/journal.pone.0128073 (PMC4447423; doi:10.1371/journal.pone.0128073)

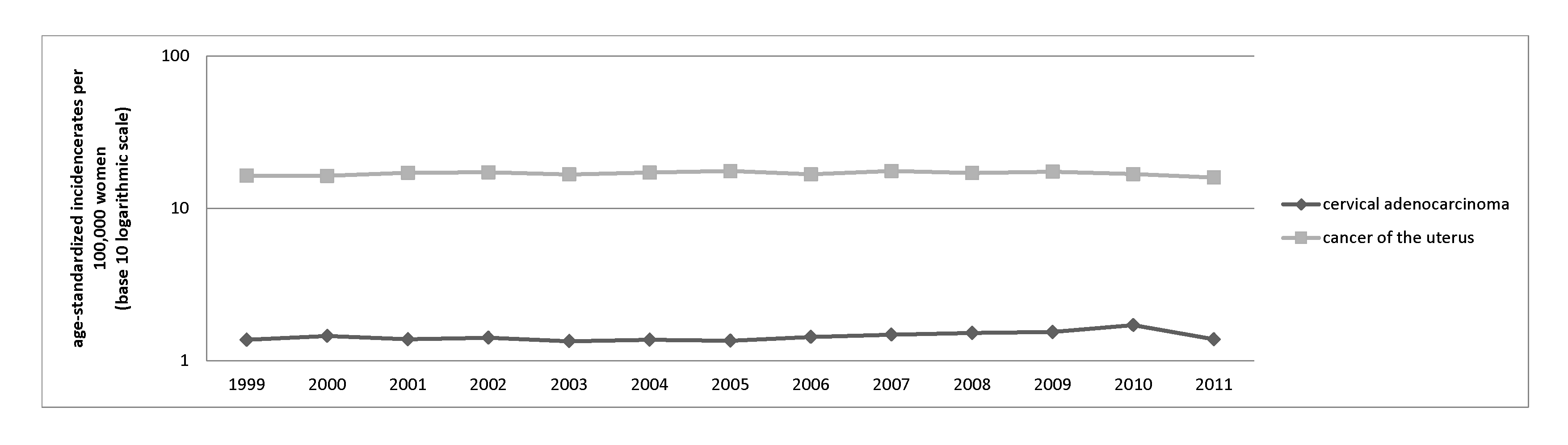

Supplement: S1 Fig — Annual age-standardized incidence rates per 100,000 women (old European Standard on a base-10 logarithmic scale) of cervical adenocarcinoma and cancer of the uterus, 1999–2011. (TIF) [file pone.0128073.s001.tif]
